# N^6^-methyladenosine (m^6^A) methyltransferase METTL3 regulates sepsis-induced myocardial injury through IGF2BP1/HDAC4 dependent manner

**DOI:** 10.1038/s41420-022-01099-x

**Published:** 2022-07-15

**Authors:** Hao Shen, Keliang Xie, Miaomiao Li, Qianyu Yang, Xiaoye Wang

**Affiliations:** 1 Department of Intensive Care Unit, Tianjin Medical University General Hospital, Tianjin, 300052 China; 2 Department of Pediatric surgery, Tianjin Children’s Hospital, Tianjin, 300074 China

**Keywords:** Epigenomics, Cardiac hypertrophy

## Abstract

Recent studies have identified that N^6^-methyladenosine (m^6^A) extensively participates in the myocardial injury pathophysiological process. However, the role of m^6^A on sepsis-induced myocardial injury is still unclear. Here, we investigated the functions and mechanism of m^6^A methyltransferase METTL3 for septic myocardial injury. Results illustrated that the m^6^A modification level and METTL3 up-regulated in the lipopolysaccharide (LPS)-induced cardiomyocytes (H9C2 cells). Methylated RNA immunoprecipitation sequencing (MeRIP-Seq) revealed the m^6^A profile of the septic myocardial injury cellular model. Functionally, METTL3 knockdown repressed the inflammatory damage of cardiomyocytes induced by LPS. Mechanistically, we found that HDAC4 had remarkable m^6^A modification sites on its 3’-UTR genome, acting as the downstream target of METTL3. Besides, m^6^A reader IGF2BP1 recognized the m^6^A modification sites on HDAC4 mRNA and enhanced its RNA stability. In conclusion, the findings illustrated a role of METTL3/IGF2BP1/m^6^A/HDAC4 axis on sepsis-induced myocardial injury, which might provide novel therapeutic strategy for septic myocardial injury.

## Abstract (web summary)

METTL3 promotes LPS-induced cardiomyocytes inflammatory damage via IGF2BP1/m6A/HDAC4 axis.

## Graphical abstract

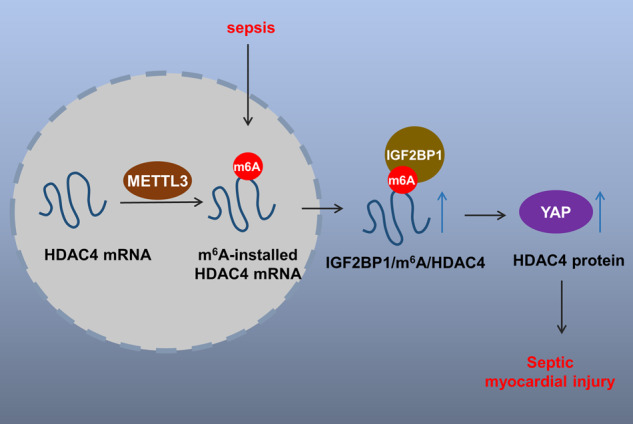

## Introduction

Sepsis is a common clinical complication and considered to be an abnormal inflammatory response caused by infection, which subsequently leads to multiple organ failure [1, 2]. In the pathophysiology of sepsis, aberrant inflammation and immune response can aggravate the sepsis, leading to shock or myocardial dysfunction [3, 4]. Sepsis-induced myocardial injury is a critical factor in the poor clinical outcome of patients with sepsis. The heart is one of the most vulnerable target organs in sepsis, presenting as cardiomyocytes degeneration and necrosis, and myocardial systolic and diastolic function dysfunction [5, 6]. The pathophysiology of sepsis-induced myocardial injury is complex, involving both the pathogen and the host immune system. At the present time, the mechanism of septic myocardial injury remains largely unclear and the effective therapeutic strategy for septic myocardial injury has not yet been structured.

N^6^-methyladenosine (m^6^A) acts as a post-transcriptional regulatory mark in multiple RNAs, including messenger RNAs (mRNAs), transfer RNAs (tRNAs), long noncoding RNA (lncRNAs) [7], small nuclear RNA et al [8–10]. It has been identified that the m^6^A RNA modification was installed by methyltransferases (writers), such as methyltransferaselike 3 (METTL3), METTL14 and METTL16. A novel writer METTL16 acts against non-coding RNA [7, 10, 11]. Besides, the m^6^A RNA modification was uninstalled by demethylase (eraser), such as ALKBH5 and FTO [12, 13]. In addition, the biological functions of m^6^A are mediated via reader protein by recognizing the m^6^A site, such as the YT521-B homology (YTH) family (YTHDFs).

Emerging evidence suggests the critical roles of m^6^A on cardiac pathophysiological process, such as heart failure progression [14], cardiac homeostasis and hypertrophy [15], cardiac cellular growth [16] and histone H3 trimethylation [17]. Besides, the functions of m^6^A have been identified in the myocardial damages. For example, in myocardial injury, METTL3 deficiency contributes to heart regeneration via METTL3-pri-miR-143-(miR-143)-Yap/Ctnnd1 [18]. Here, our research found that METTL3 and m^6^A modification level up-regulated in the LPS-induced cardiomyocytes. Besides, METTL3 exerted its regulation via m^6^A-dependent HDAC4 manner. In summary, METTL3-mediated m^6^A modification is expected to be a therapeutic target for septic myocardial injury.

## Results

### The m^6^A profile in LPS-administrated cardiomyocytes

**Fig. 1:**
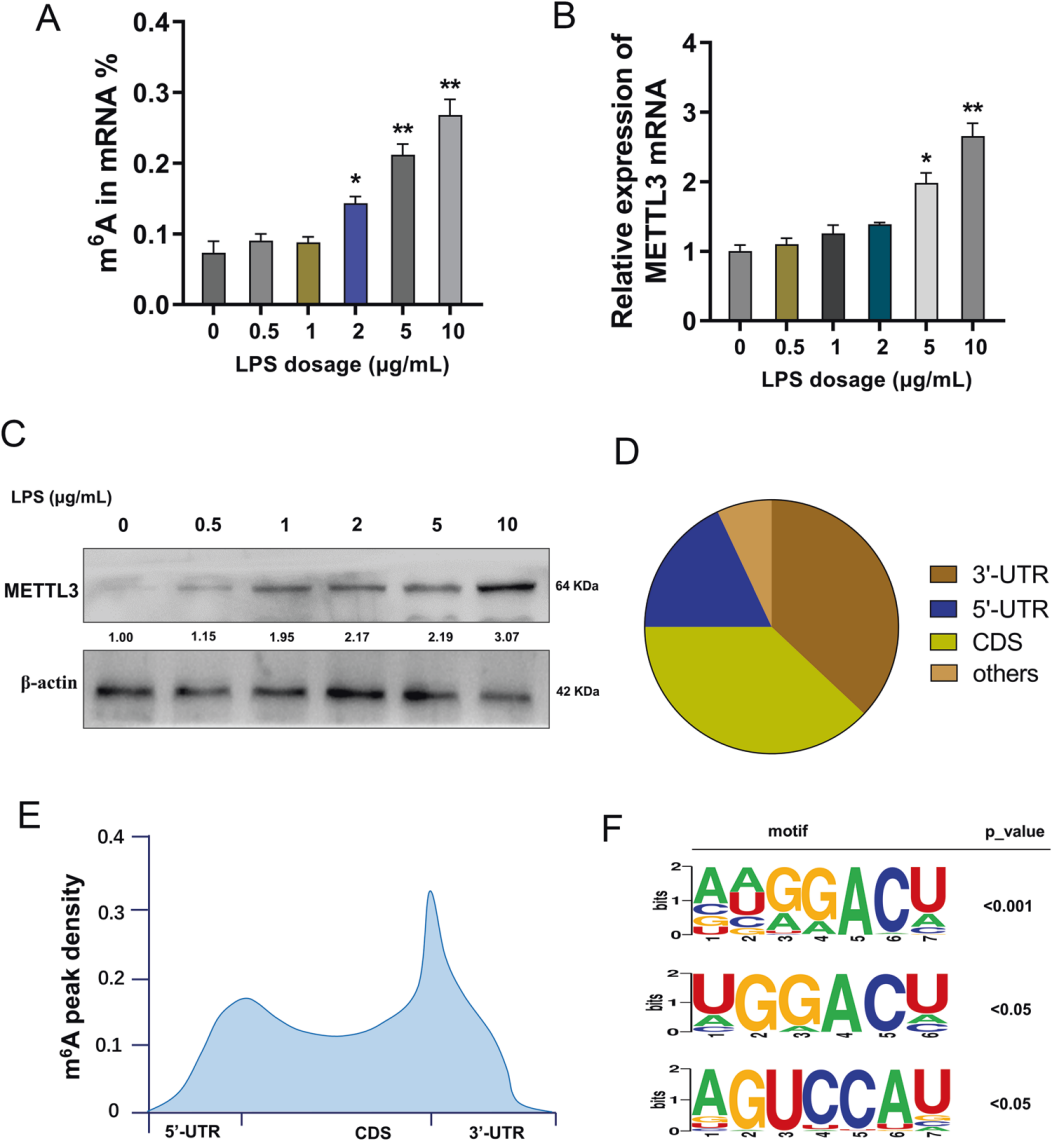
The m^6^A profile in LPS-administrated cardiomyocytes. **A** Cardiomyocytes (H9C2) was induced by LPS (10 μg/mL) to construct myocardial cell injury for septic myocardial cell injury investigation. **B** RT-PCR indicated the mRNA level of m^6^A methyltransferase METTL3 in cardiomyocytes with the increasing doses of LPS (0, 0.5, 1, 2, 5, 10 μg/mL) for 24 h. **C** Western blot analysis for the protein level in cardiomyocytes with the increasing doses of LPS (0, 0.5, 1, 2, 5, 10 μg/mL) for 24 h. **D** The m^6^A modification distribution in myocardial cell injury cardiomyocytes revealed by methylated RNA immunoprecipitation sequencing (MeRIP-Seq). **E** m^6^A metagene showed the m^6^A peak density in integral genome. **F** The candidate motifs. Biological experiments were performed in triplicate. Data were exhibited as Mean ± Standard Deviation (SD). **p < 0.01, *p < 0.05.

To investigate the potential function of m^6^A in the septic myocardial cell injury, cardiomyocytes (H9C2) was induced by LPS (10 μg/mL) to construct myocardial cell injury. In the LPS-administrated cardiomyocytes, the m^6^A modification level increased in a concentration-dependent manner (Fig. 1A). Moreover, the m^6^A methyltransferase METTL3 expression increased with the increasing doses of LPS (0, 0.5, 1, 2, 5, 10 μg/mL) for 24 h for sepsis cellular model construction, including mRNA (Fig. 1B) and protein (Fig. 1C). Methylated RNA immunoprecipitation sequencing (MeRIP-Seq) revealed the m^6^A modification distribution in myocardial cell injury cardiomyocytes, including 3’-UTR, 5’-UTR and CDS (Fig. 1D). Besides, m^6^A metagene showed the m^6^A peak density in integral genome (Fig. 1E). The candidate motifs were also screened for the following analysis (Fig. 1F). In summary, the MeRIP-Seq recovered the m^6^A profile in LPS-administrated cardiomyocytes.

### METTL3 knockdown repressed the inflammatory damage of cardiomyocytes induced by LPS

**Fig. 2:**
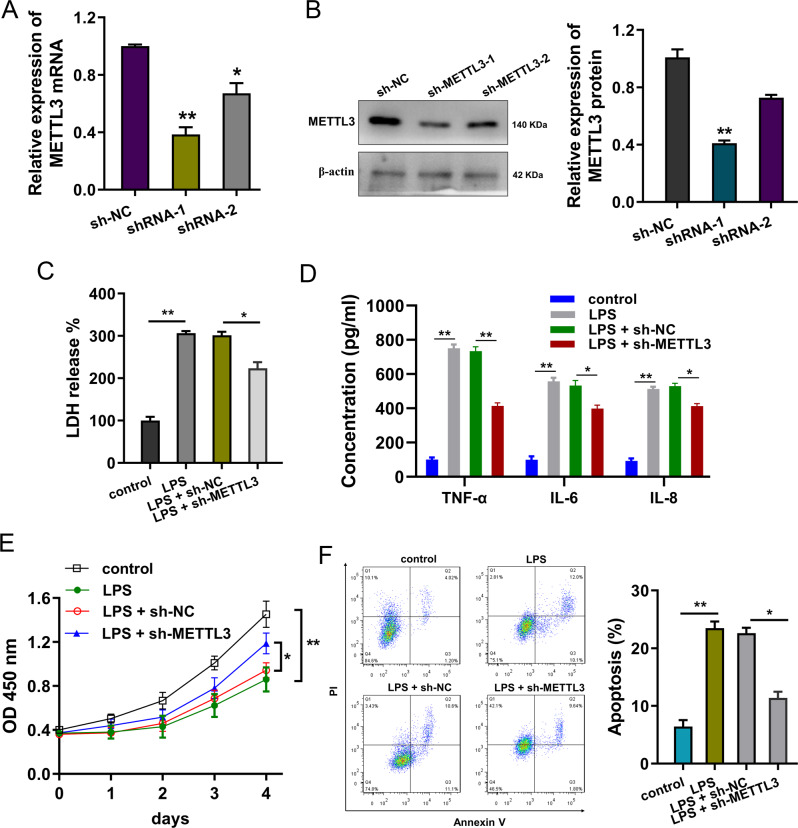
METTL3 knockdown repressed the inflammatory damage of cardiomyocytes induced by LPS. **A** The silencing efficiency was detected using RT-qPCR and (**B**) western blot for cardiomyocytes transfected with METTL3 knockdown (sh-METTL3) or controls (sh-NC). **C** The lactate dehydrogenase (LDH) release analysis was performed using microplate reader, including control, LPS, LPS + sh-NC, LPS + sh-METTL3. **D** ELISA analysis was performed to detect the levels of proinflammatory cytokines (interleukin (IL)-6, IL-8, and tumor necrosis factor-alpha (TNF-α)) for cardiomyocytes (H9C2), including control, LPS, LPS + sh-NC, LPS + sh-METTL3. **E** Proliferative ability using CCK-8 assay for the proliferation ability of cardiomyocytes, including control, LPS, LPS + sh-NC, LPS + sh-METTL3. **F** Flow cytometry for apoptosis analysis revealed the apoptosis of cardiomyocytes transfected with METTL3 knockdown (sh-METTL3) or controls (sh-NC) upon LPS administration. Biological experiments were performed in triplicate. Data were exhibited as Mean ± Standard Deviation (SD). **p < 0.01, *p < 0.05.

To investigate the function of METTL3 in myocardial cell injury, METTL3 knockdown was constructed. The silencing efficiency was detected using RT-PCR (Fig. 2A) and western blot (Fig. 2B). The LDH release analysis found that LPS administration promoted the LDH release and METTL3 silencing repressed the LDH release (Fig. 2C). ELISA analysis revealed that LPS administration up-regulated the levels of proinflammatory cytokines, including interleukin (IL)-6, IL-8, and tumor necrosis factor-alpha (TNF-α), while METTL3 silencing reduced the levels of proinflammatory cytokines (Fig. 2D). Proliferative ability assay indicated that LPS administration repressed the proliferation and METTL3 silencing up-regulated the proliferation ability (Fig. 2E). Flow cytometry for apoptosis analysis revealed that LPS administration induced the apoptosis of cardiomyocytes and then METTL3 silencing decreased the apoptotic rate (Fig. 2F). Taken together, these findings revealed that METTL3 knockdown repressed the inflammatory damage of cardiomyocytes induced by LPS.

### HDAC4 acted as a target of METTL3 in cardiomyocytes

**Fig. 3:**
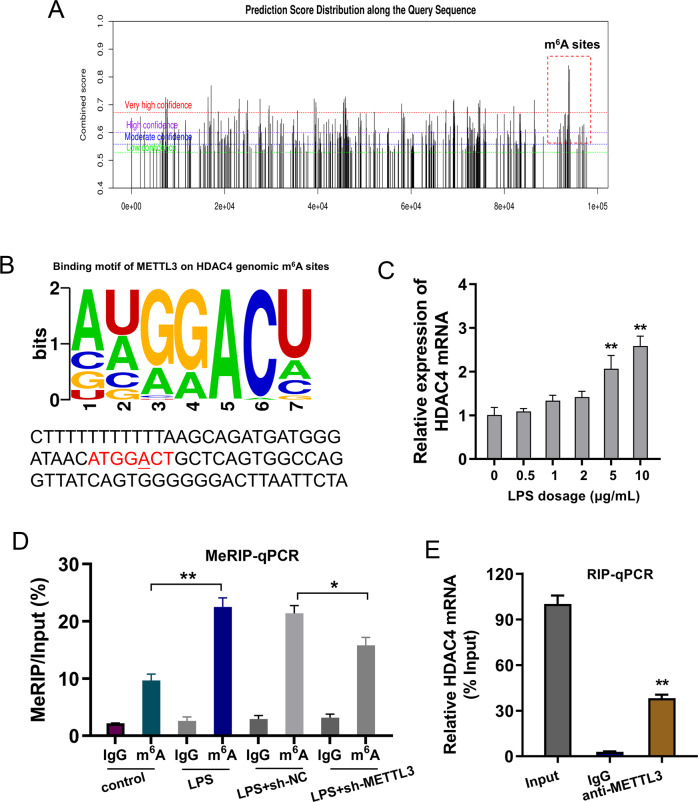
HDAC4 acted as a target of METTL3 in cardiomyocytes. **A** The predictive tools using sequence-based N^6^-methyladenosine (m^6^A) modification site predictor (SRAMP, http://www.cuilab.cn/sramp) revealed the potential m^6^A modification sites on HDAC4 genome. **B** The binding motif of METTL3 on HDAC4 m^6^A site was GGACU sequences. **C** RT-PCR detected the HDAC4 mRNA was detected in cardiomyocytes with the increasing doses of LPS (0, 0.5, 1, 2, 5, 10 μg/mL). **D** Knockdown of METTL3 decreased the m^6^A methylation level of HDAC4 mRNA using m^6^A MeRIP-qPCR analysis. **E** RIP assays reflected the interaction of METTL3 and HDAC4 mRNA in cardiomyocytes. Relative enrichment of HDAC4 mRNA associated with METTL3 relative to input was calculated. IgG antibody acted as a control. Biological experiments were performed in triplicate. Data were exhibited as Mean ± Standard Deviation (SD). **p < 0.01, *p < 0.05.

The predictive tools using sequence-based N6-methyladenosine (m^6^A) modification site predictor (SRAMP, http://www.cuilab.cn/sramp) revealed that there were several potential m^6^A modification sites on HDAC4 genome (Fig. 3A). The binding sites motif of METTL3 on HDAC4 m^6^A site showed the GGACU sequences (Fig. 3B). The HDAC4 mRNA was detected in cardiomyocytes with the increasing doses of LPS (0, 0.5, 1, 2, 5, 10 μg/mL), showing the high level of HDAC4 mRNA upon LPS administration (Fig. 3C). MeRIP-qPCR analysis revealed that LPS administration up-regulated the m^6^A modification level of HDAC4 mRNA, while METTL3 knockdown reduced the m^6^A enrichment (Fig. 3D). The interaction within METTL3 and HDAC4 mRNA using RNA immunoprecipitation assay (RIP-qPCR) showed that METTL3 interacted with HDAC4 mRNA via molecular bindings (Fig. 3E). Taken together, these findings suggested that HDAC4 acted as a target of METTL3 in cardiomyocytes.

### METTL3/IGF2BP1 enhanced the stability of HDAC4 mRNA

**Fig. 4:**
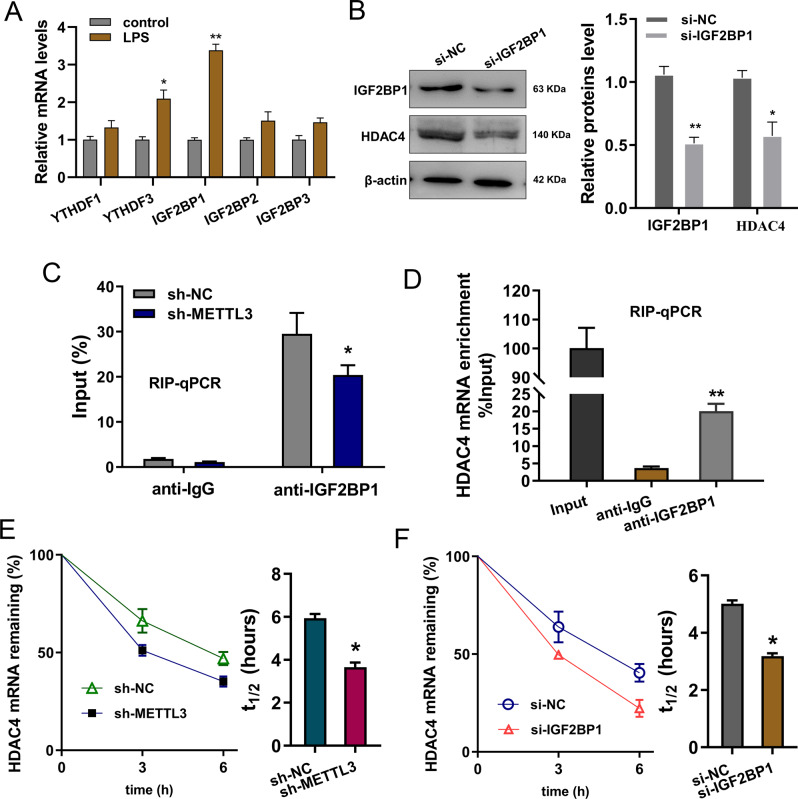
METTL3/IGF2BP1 enhanced the stability of HDAC4 mRNA. **A** RT-PCR detected the expression of candidate m^6^A readers (YTHDF1, YTHDF2, IGF2BP1, IGF2BP2, IGF2BP3) in myocardial injury cardiomyocytes (H9C2) induced by LPS (10 μg/mL). **B** Western blot analysis detected the level of HDAC4 protein. **C** RNA immunoprecipitation analysis (RIP-qPCR) showed the enrichment of HDAC4 mRNA precipitated by anti-IGF2BP1 or anti-IgG in cardiomyocytes transfected with shRNA-METTL3. **D** RIP-qPCR was performed to detected the HDAC4 mRNA level precipitated by anti-IGF2BP1 or anti-IgG in cardiomyocytes. **E** shRNA-METTL3 or **F** si-IGF2BP1 was transfected into Act D treated cardiomyocytes and the HDAC4 mRNA level was detected using RT-qPCR. Biological experiments were performed in triplicate. Data were exhibited as Mean ± Standard Deviation (SD). **p < 0.01, *p < 0.05.

Investigation analysis found that, among several candidate m^6^A readers (YTHDF1, YTHDF2, IGF2BP1, IGF2BP2, IGF2BP3), IGF2BP1 showed a remarkable high-expression in LPS administration (Fig. 4A). Moreover, we found that IGF2BP1 knockdown reduced the expression level of HDAC4 protein (Fig. 4B). RNA immunoprecipitation analysis (RIP-qPCR) revealed that the silencing of METTL3 intercepted the interaction within IGF2BP1 and HDAC4 mRNA (Fig. 4C). Besides, RIP-qPCR illustrated that IGF2BP1 closely combined with HDAC4 mRNA in cardiomyocytes treated with LPS (Fig. 4D). RNA stability analysis found that knockdown of IGF2BP1 and METTL3 both reduced the HDAC4 mRNA level upon Act D treatment (Fig. 4E, F). In summary, these findings revealed that METTL3/IGF2BP1 enhanced the stability of HDAC4 mRNA.

### METTL3/IGF2BP1/HDAC4 axis regulates the inflammatory damage of cardiomyocytes induced by LPS

**Fig. 5:**
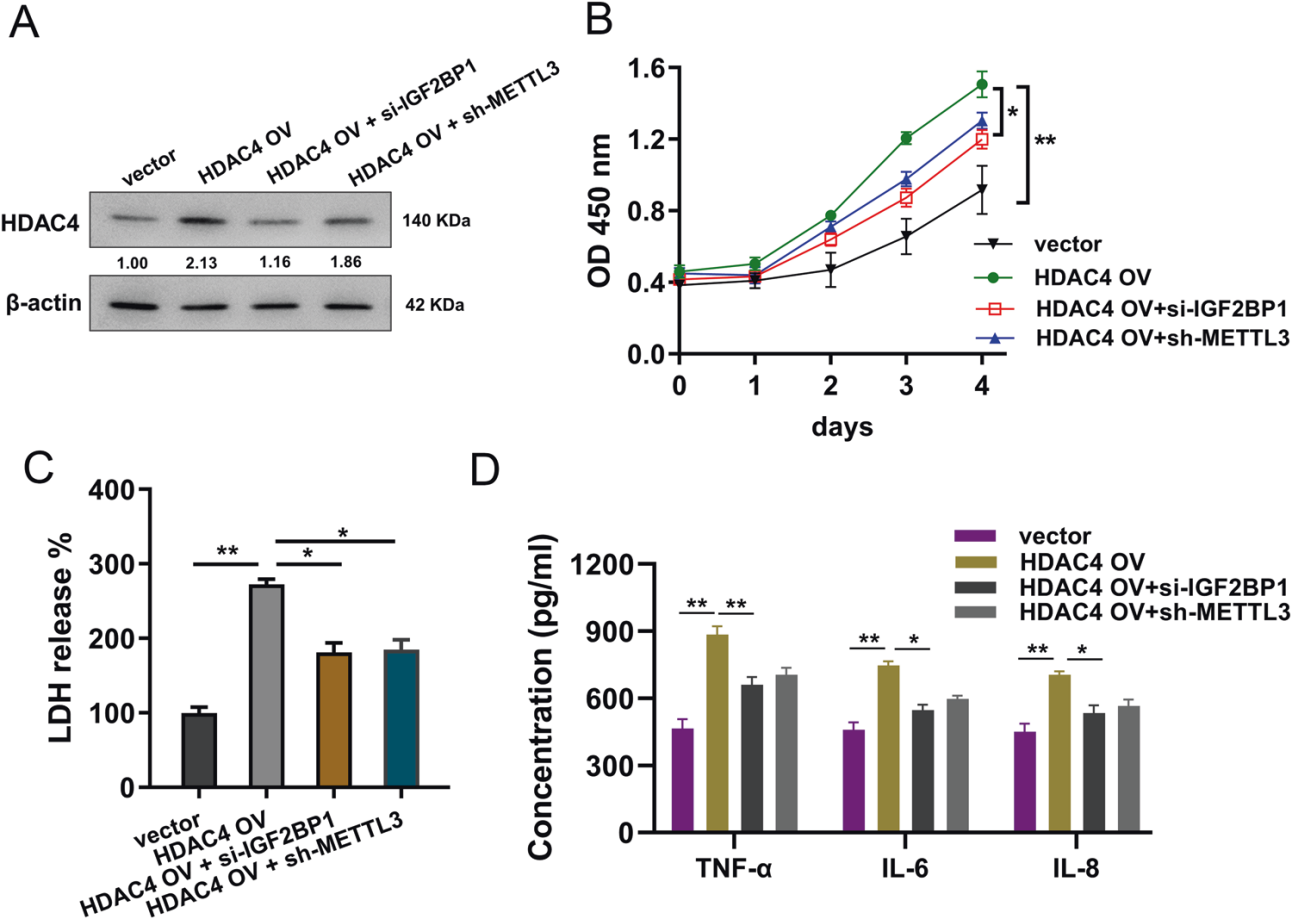
METTL3/IGF2BP1/HDAC4 axis regulates the inflammatory damage of cardiomyocytes induced by LPS. **A** Western blot analysis detected the HDAC4 protein level in cardiomyocytes (H9C2) transfected with HDAC4 overexpression (HDAC4 OV) or IGF2BP1 knockdown (si-IGF2BP1) or METTL3 knockdown (sh-METTL3) respectively. **B** CCK-8 analysis was performed to detect the proliferative ability of cardiomyocytes. **C** ELISA analysis detected the levels of proinflammatory cytokines, including interleukin (IL)-6, IL-8, and tumor necrosis factor-alpha (TNF-α). Biological experiments were performed in triplicate. Data were exhibited as Mean ± Standard Deviation (SD). **p < 0.01, *p < 0.05.

To identify the role of METTL3/IGF2BP1 on cardiomyocytes’ inflammatory damage, rescue assays were performed using co-transfection of HDAC4 overexpression and METTL3/IGF2BP1 knockdown. Western blot analysis found that HDAC4 overexpression transfection could be reduced by IGF2BP1 knockdown or METTL3 knockdown (Fig. 5A). The proliferative analysis found that HDAC4 overexpression augmented the proliferation of cardiomyocytes, while IGF2BP1 knockdown or METTL3 knockdown could ameliorate the proliferation (Fig. 5B). The LDH release analysis showed that HDAC4 overexpression promoted the LDH release, and IGF2BP1 knockdown or METTL3 knockdown recovered the LDH release (Fig. 5C). ELISA analysis revealed that HDAC4 overexpression up-regulated the levels of proinflammatory cytokines, including interleukin (IL)-6, IL-8, and tumor necrosis factor-alpha (TNF-α), while IGF2BP1 knockdown or METTL3 knockdown recovered the levels of proinflammatory cytokines (Fig. 5D). In summary, these findings revealed that METTL3/IGF2BP1/HDAC4 axis regulated the inflammatory damage of cardiomyocytes induced by LPS.

## Discussion

Clinically, sepsis acts as a major threat with high mortality rate for patients. The response of host immune system to pathogen infection is a key biological process in the occurrence and development of sepsis. The mortality rate of septic patients complicated by cardiac dysfunction is 70−90%, which is significantly higher than that of patients with cardiac dysfunction [19, 20]. The septic stimulation on cardiomyocytes could cause the myocardium injury, which acts as a key factor in the development of organ dysfunction, and also an important factor for the next treatment of septis patients. However, there is still no consensus on the exact definition of sepsis myocardial injury.

According to the current research progress, it is generally believed that sepsis myocardial injury is related to the interaction of multiple factors, such as Toll-like receptors, mitochondrial damage, cytokines, apoptosis, calcium imbalance and nitric oxide imbalance [21, 22]. Lipopolysaccharide (LPS) is a critical component of the outer membrane of gram-negative bacteria, which could cause septic or infectious cardiac dysfunctions or cardiac cell death by stimulating inflammation [23]. Here, we constructed the sepsis myocardial injury model using LPS-induced cardiomyocytes (H9C2 cells).

In septic cardiomyocytes, the expression or enrichment of m^6^A and METTL3 increased, suggesting the potential roles and regulation of m^6^A modification in sepsis-induced myocardial injury. Besides, the knockdown of METTL3 repressed the inflammatory damage of cardiomyocytes induced by LPS, including interleukin (IL)-6, IL-8, and tumor necrosis factor-alpha (TNF-α). Thus, we could derive the conclusion that METTL3 might act as an essential regulator in sepsis-induced myocardial injury, which is dependent on the m^6^A modification manner.

As regarding to the molecular mechanism by which METTL3 accelerates septic myocardial injury, we investigated the interacted elements of METTL3. Among several candidate m^6^A readers (YTHDF1, YTHDF2, IGF2BP1, IGF2BP2, IGF2BP3), IGF2BP1 showed a remarkable high expression in LPS administration. Importantly, METTL3 could closely combine with IGF2BP1, which was identified by RNA Immunoprecipitation assay. Next, HDAC4 gene was discovered to be the downstream of IGF2BP1. In summary, METTL3 installed the m^6^A modification on HDAC4 mRNA, and then IGF2BP1 was responsible for the recognition and RNA stability maintain for HDAC4 mRNA. Given that histone deacetylases (HDACs) have been found to regulate the myocardial injury [24], the potential roles of HDAC4 are critical for septic myocardial injury [25]. Here, our findings revealed that HDAC4 overexpression promoted the cardiomyocytes’ inflammatory damage of cardiomyocytes induced by LPS.

The emerging roles of m^6^A modification on sepsis-induced organ dysfunction have been gradually verified [26–28]. For example, in lipopolysaccharide (LPS)-induced endotoxemia on myocardial inflammation, the m^6^A-RNA methylation level and inflammatory cytokine genes increased, while FTO knockdown mimicked the effects [29]. Another example, in septic macrophage responses, LPS treatment induces Socs1 mRNA m^6^A methylation and METTL14 depletion mitigates the m^6^A methylation of Socs1 mRNA to reduce YTHDF1 binding ability to Socs1 mRNA the m^6^A sites [30]. Thus, the in-depth study targeting m^6^A modification could provide novel insight for septic myocardial injury.

In conclusion, these findings revealed that m^6^A methyltransferase METTL3 participated in the myocardial injury induced by sepsis via the IGF2BP1/HDAC4 axis. METTL3/IGF2BP1 enhanced the stability of HDAC4 mRNA, thereby accelerating the inflammatory damage of cardiomyocytes induced by LPS. These findings suggested a therapeutic target for sepsis-induced myocardial injury.

## Materials and methods

### Cells culture and administration

Rat cardiomyocyte (H9C2) cells were provided from ScienCell (Catalog #R6200). H9C2 cells were incubated in Dulbecco’s modified Eagle’s medium (DMEM) supplemented with 10% fetal bovine serum (FBS, Gibco, Gran Island, NY, USA) and 1% penicillin-streptomycin (Solarbio) in 37 °C incubator with 5% CO_2_. Every two days, medium was refreshed and when reaching 80% confluence, H9C2 cells were treated with increasing doses of LPS (0, 0.5, 1, 2, 5, 10 μg/mL) for 24 h for sepsis cellular model construction.

### Transfection

To generate stable silenced METTL3 expression, vectors containing shRNA were inserted into PLKO.1 (Ribobio, Guangzhou, China). To knockdown IGF2BP1 expression, siRNAs targeting IGF2BP1 were constructed. The negative vector, si-NC and sh-NC, acted as the control groups. All constructs were confirmed by sequencing and synthesized by RiboBio (Guangzhou, China). After 24 hours of LPS (10 μg/mL) administration, cardiomyocytes (H9C2) were transfected with oligonucleotides as protocols’ instruction. Cardiomyocytes were planted in six-well plates 24 h prior to shRNA or siRNA transfection with 50−60% confluence, and then mixed with Lipofectamine 2000 (Invitrogen, Carlsbad, CA, USA) according to the manufacture instructions. The sequences that were used are shown in Table S1.

### RNA extraction and real‑time quantitative PCR (RT‑qPCR)

Total RNA was extracted using RNA extraction kit (QIAGEN, No.77064) according to the manufacture instructions. cDNA was synthesized using a reverse transcription kit (Promega, Madison, USA) according to the manufacturer’s instructions. PCR was conducted using the SYBR Green Master Mix (SYBR Green PCR kit (TaKaRa, Dalian, China) on Applied Biosystems 7300). After the reactions, relative gene expression levels were calculated using the formula 2^−ΔΔCt.^. The primer sequences used were indicated in Supplementary Table S1.

### Western blot

Cardiomyocytes were lysed with RIPA buffer (cat: P0013B, Beyotime Biotech, Shanghai, China). The supernatants were resolved in sodium dodecyl sulfate-polyacrylamide gel electrophoresis (SDS-PAGE) and transferred onto polyvinylidene fluoride (PVDF) membranes (cat: IPVH00010, Millipore, MA, USA). Membranes were incubated with anti-METTL3 (1:1000, ab195352, Abcam), anti-IGF2BP1 (1:1000, ab184305, Abcam), anti-HDAC4 (1:1000, ab12174, Abcam) and anti-β-actin (ab5694, 1:1000, Abcam) overnight at 4 °C. Afterward, the membranes were incubated with HRP-conjugated goat antirabbit IgG (ab205718, 1:2000, Abcam) and visualized using an enhanced chemiluminescence (ECL) methods. Signal density was analyzed by Image J software and β-actin as an internal reference.

### Methylated RNA immunoprecipitation sequencing (MeRIP-Seq)

The MeRIP-Seq was performed as described previously [31]. In brief, total RNA was extracted from cells and then poly-A-purified RNA was fragmented and incubated with m^6^A primary antibody. The mixture was immunoprecipitated by incubation with Protein A beads. The captured RNA was washed for three times, and the eluted m^6^A-nucleotide solution and purified. Input immunoprecipitation samples and m^6^A IP samples were used for library generation and then evaluated with BioAnalyzer 2100 system (Agilent Technologies). Library sequencing was performed on illumina Hiseq instrument with 150 bp paired-end reads.

### Proliferation assay

Proliferative viability assay was performed using CCK-8 assays. In brief, 5×10^3^ cells were seeded in 96-well plate overnight and treated with inhibitors at indicated times. Those cells were incubated with cell counting kit-8 (CCK-8) reagent (CK04, Dojindo Laboratories, Kumamoto, Japan) was added to the plate (10 mL/well). The optical density (OD) value was measured at OD 450 nm and analyzed by GraphPad Prism 8.0.

### Enzyme-linked immunosorbent assay (ELISA) and lactate dehydrogenase (LDH) release

The TNF-α, IL-8, and IL-6 levels were determined with the rat TNF-α ELISA kit (Solarbio), IL-8 ELISA kit (Solarbio) and IL-6 ELISA kit (Solarbio) according to the manufacturer’s instructions respectively. LDH release was measured using the LDH assay kit (Nanjing Jiancheng Bioengineering Institute, Nanjing, China) according to the manufacturer’s recommendations. Samples were analyzed at 450 nm wavelength by microplate reader.

### Apoptosis analysis

The apoptosis was performed using flow cytometry analysis. In brief, 1×10^6^ cells were washed with cold PBS and suspended in binding Buffer. Cells were resuspended in PBS and incubated for 40 min on ice. Cells were resuspended in 100 μl of Binding Buffer, 5 μl fluorescein isothiocyanate (FITC)-annexin V and propidium iodide (PI, 5 μl) were added to stain for 15 min at room temperature in the dark using FITC Annexin V Apoptosis Detection Kit (BD Biosciences). Lastly, the cells were analyzed by FACS Canto II flow cytometry (BD Biosciences).

### RNA m^6^A quantification

The total RNA was extracted from cardiomyocytes using TRIzol reagent (Invitrogen). In total RNA, the m^6^A levels were tested using m^6^A RNA methylation detection kit (cat. ab185912, Abcam). In brief, sample RNA (200 ng) was added into 96-well plates. 50 mL diluted capture antibody was added to each well and 100 mL diluted developer solution and termination solution on the microplate reader at 450 nm. The percentage of m^6^A in total RNA could be calculated.

### RNA immunoprecipitation (RIP)

The interaction within HDAC4 and METTL3 was identified using RIP assay with RNA-Binding Protein Immunoprecipitation Kit (cat. 17-700, Merck Millipore Darmstadt, Germany). Immunoprecipitations of endogenous HDAC4 were performed using anti-METTL3 antibody (Abcam) and anti-IGF2BP1 antibody (Abcam) overnight at 4 °C. After elution, the immunoprecipitated protein-RNA complex was analyzed by quantitative real-time polymerase chain reaction (qRT-PCR) using primers for mRNAs and normalizing to input.

### m^6^A-RNA immunoprecipitation assay (MeRIP-qPCR)

The m^6^A modification level of mRNA was detected using MeRIP-qPCR by Magna MeRIP Kit (Cat. CR203146, Millipore, Massachusetts, USA) was used according to the manufacturer’s instructions. In brief, magnetic beads (Millipore, Massachusetts, USA) were coated with anti-m^6^A antibody (No. 202003, 5 μg) (Synaptic Systems, Goettingen, Germany) or anti-IgG at room temperature for 30 min and then incubated with total RNA (50 µg) in RNase-inhibiting immunoprecipitation buffer overnight at 4 °C. After proteinase K digestion, m^6^A-bound RNA was precipitated and the m^6^A enrichment was assessed by qPCR and normalized to the input.

### RNA stability analysis

Cardiomyocytes were seeded into six-well plates at approximate 90% confluence. After transfected with shRNA or control (NC), cells were treated with 8 μg/ml actinomycin D (Act D) for 0, 3, 6 h. RNA was extracted and used for reverse transcription of qRT-PCR. The relative quantification was calculated by 2^−ΔΔCt^ method normalized to GAPDH. The half-time of HDAC4 mRNA was calculated.

### Statistical analysis

Data was analyzed using Prism 7.0 (GraphPad, La Jolla, CA, USA). All data were exhibited as the Mean ± Standard Deviation (SD) from three repetitions. Biological experiments were performed in triplicate. Difference was analyzed using t-test for two-groups or two-way analysis of variance (ANOVA) following Turkey’s post hoc test for multiple-groups. p less than 0.05 represents significant difference.

## Supplementary information

Original data file_WB blots

Fig 1 Actin blot

Fig 1 METTL3 blot

Fig 2 actin

Fig 2 METTL3

Fig 5

Table S1
