# Supplementary material for: N6-methyladenosine (m6A) methyltransferase METTL3 regulates sepsis-induced myocardial injury through IGF2BP1/HDAC4 dependent manner
Source: Cell Death Discov. 2022 Jul 15;8:322. doi: 10.1038/s41420-022-01099-x (PMC9287338; doi:10.1038/s41420-022-01099-x)

Figure 1C blot

METTL3

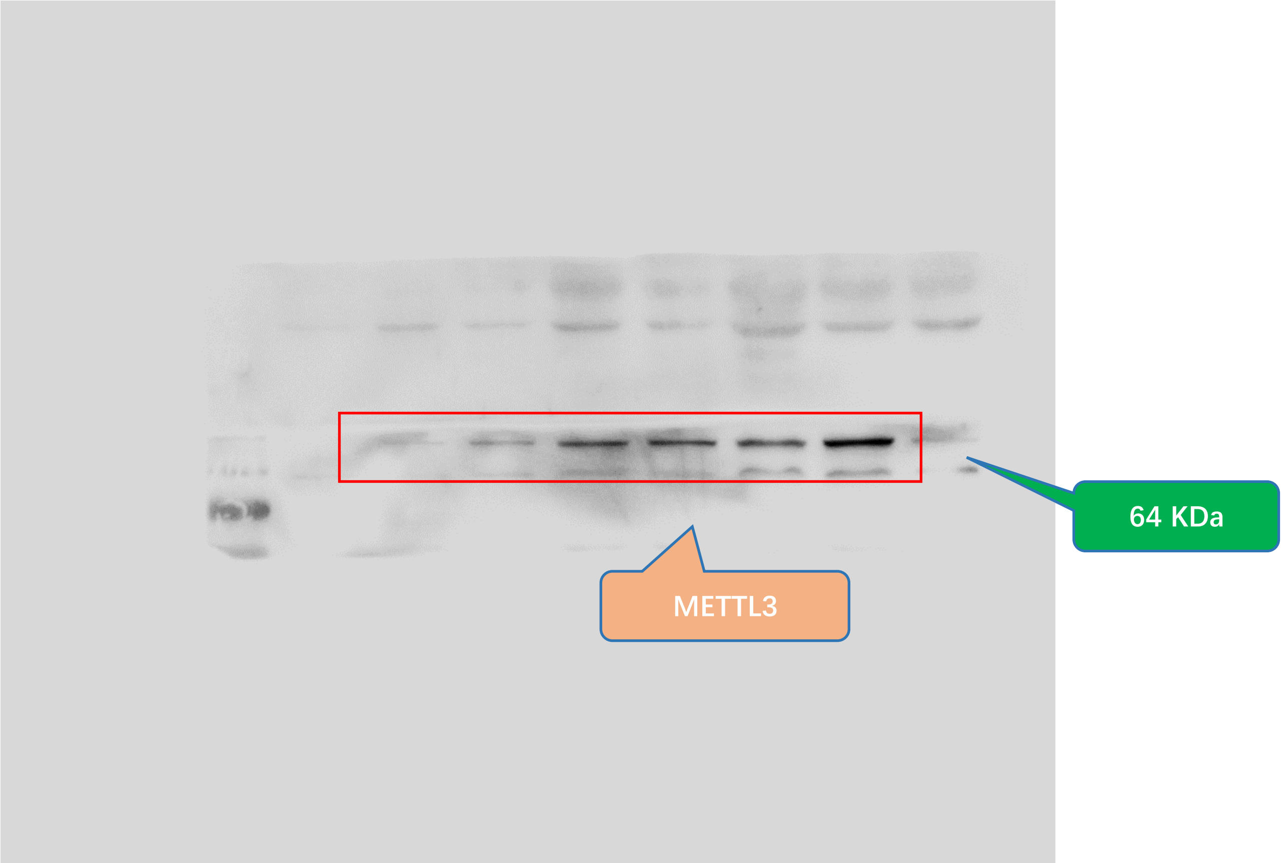

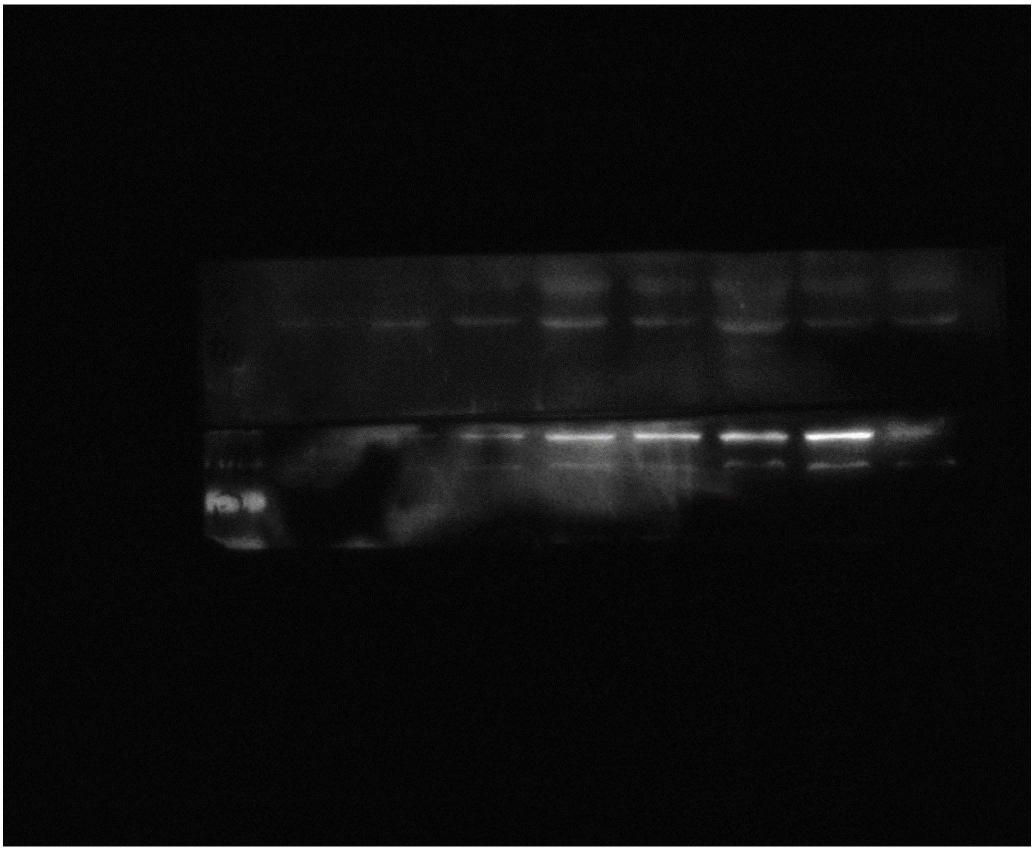

Figure 1C blot

Beta-actin

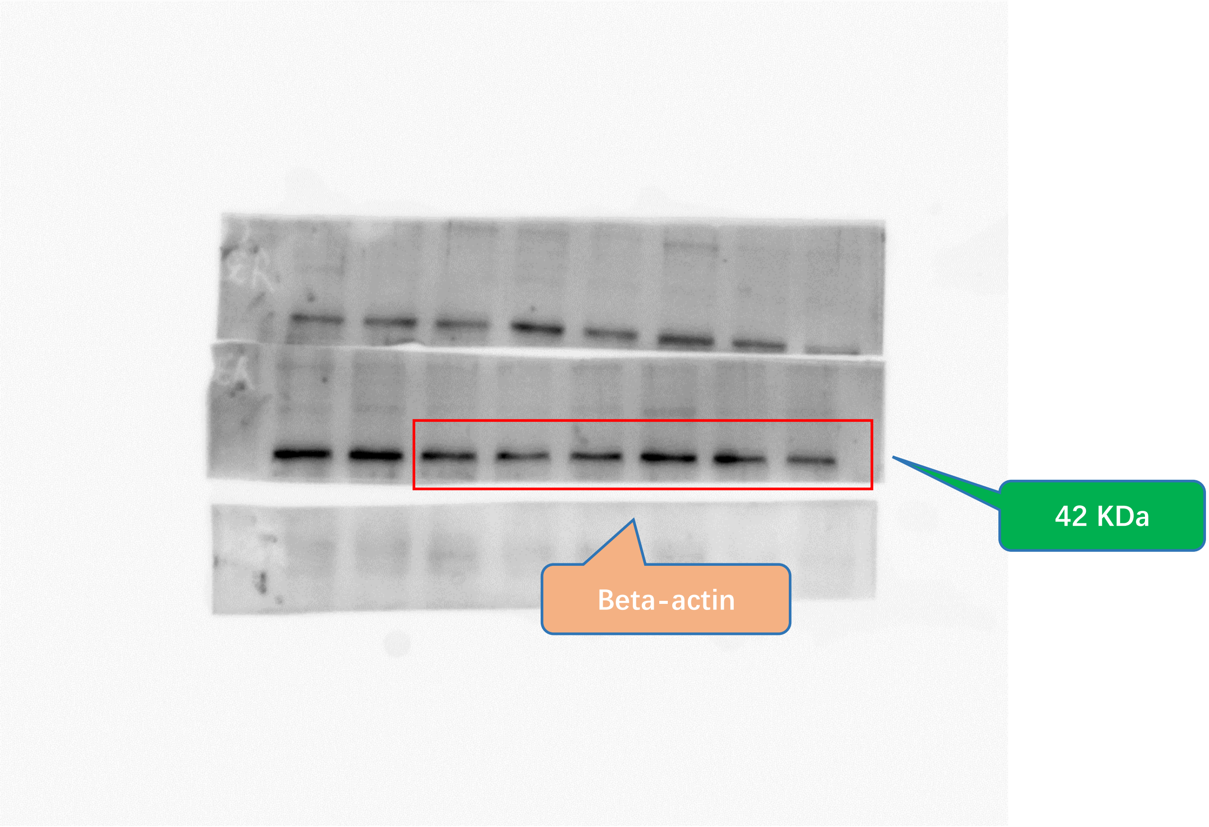

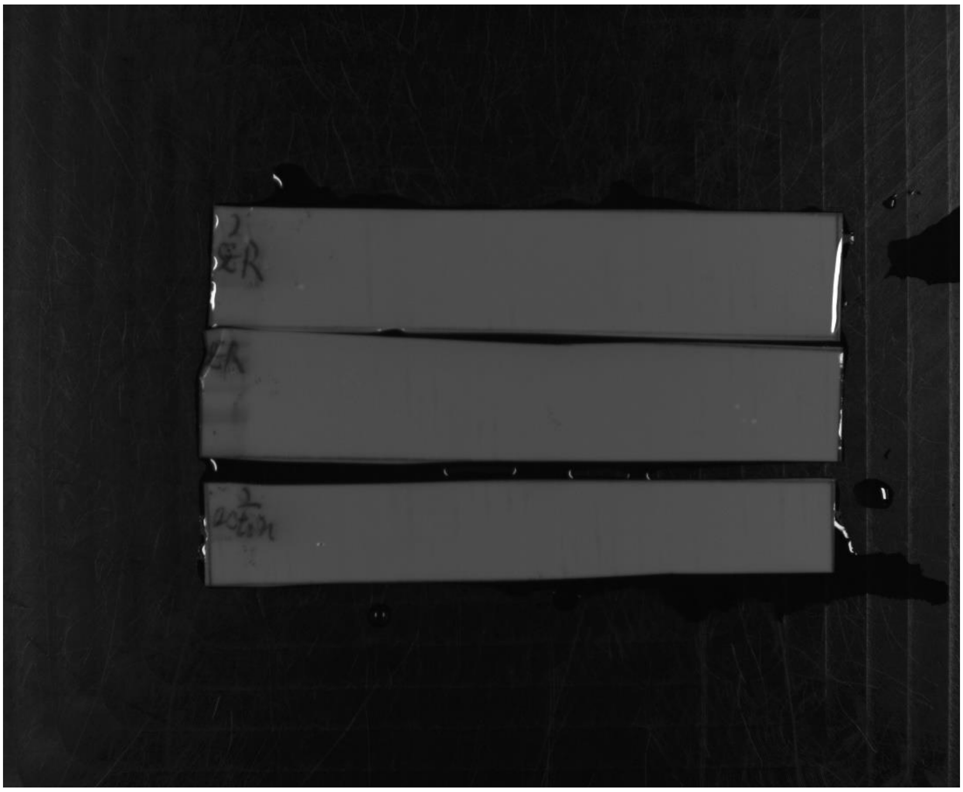

Figure 2B blot

METTL3

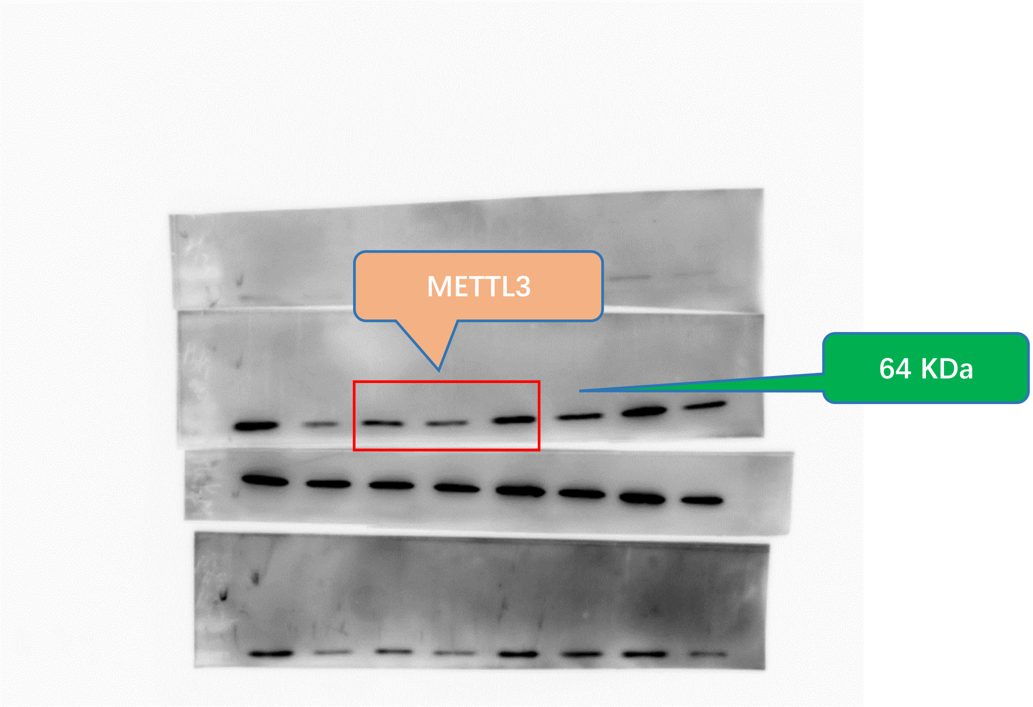

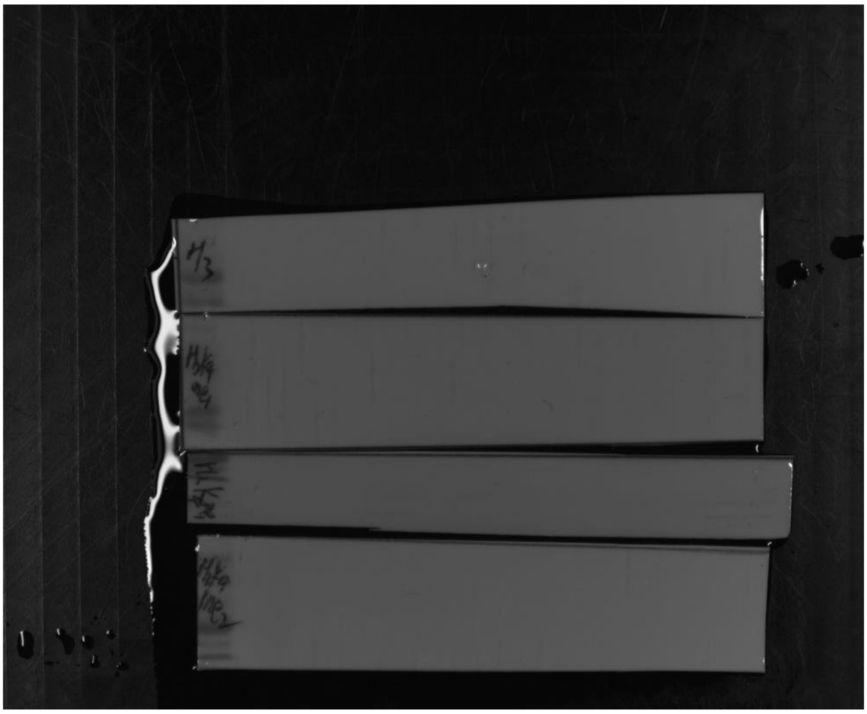

Figure 2B blot

Actin

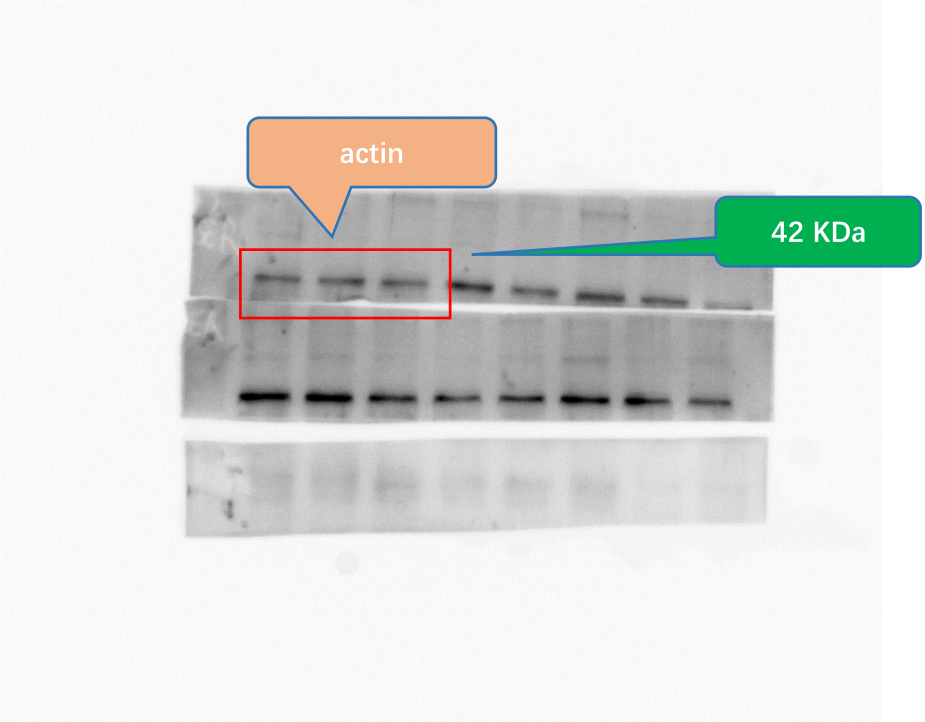

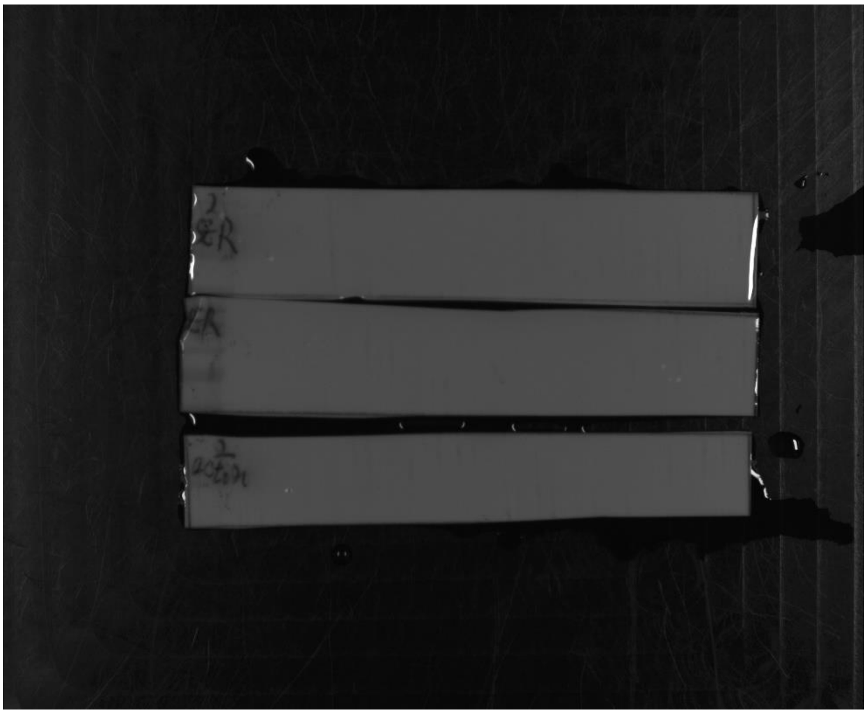

Figure 4B blot

Actin

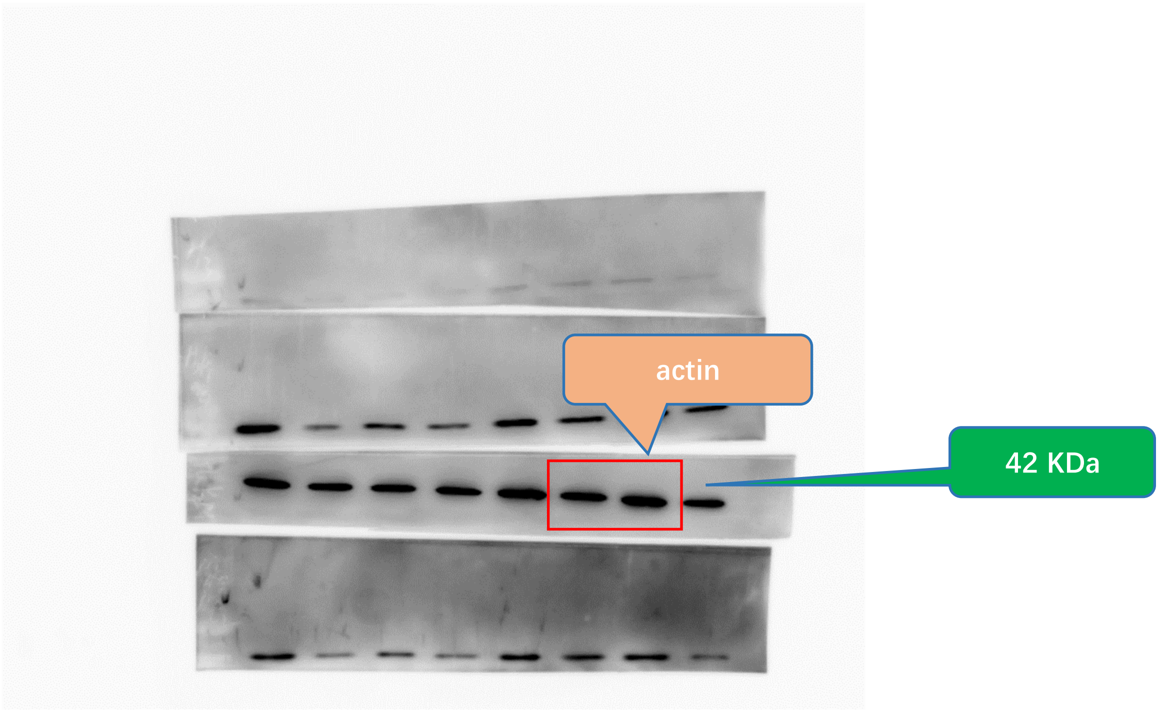

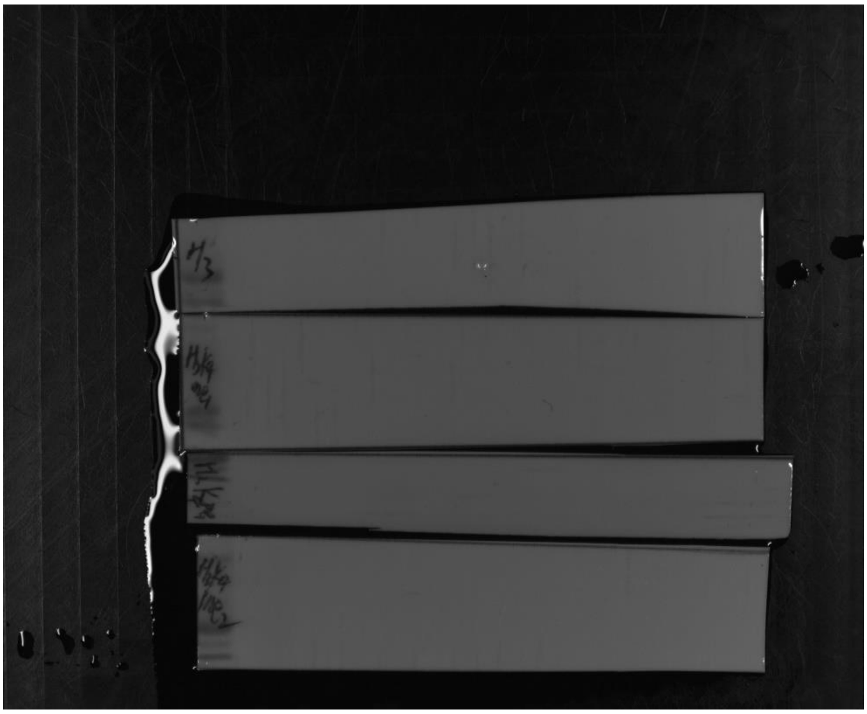

Figure 4B blot

IGF2BP1

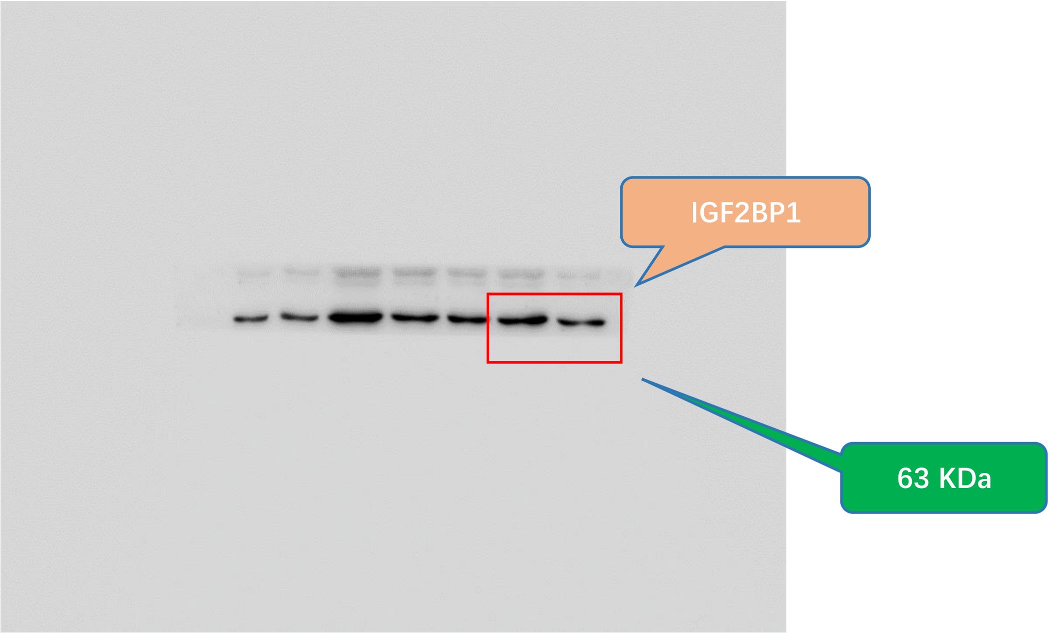

Figure 4B blot

HDAC4

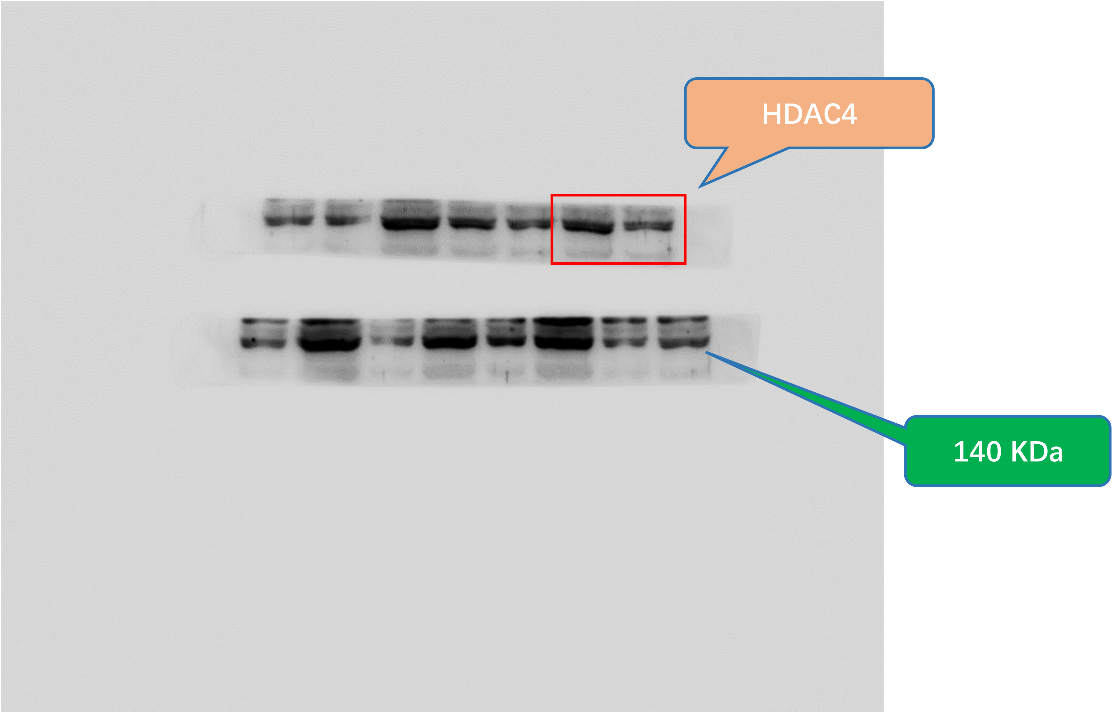

Figure 5A blot

HDAC4 and beta-actin

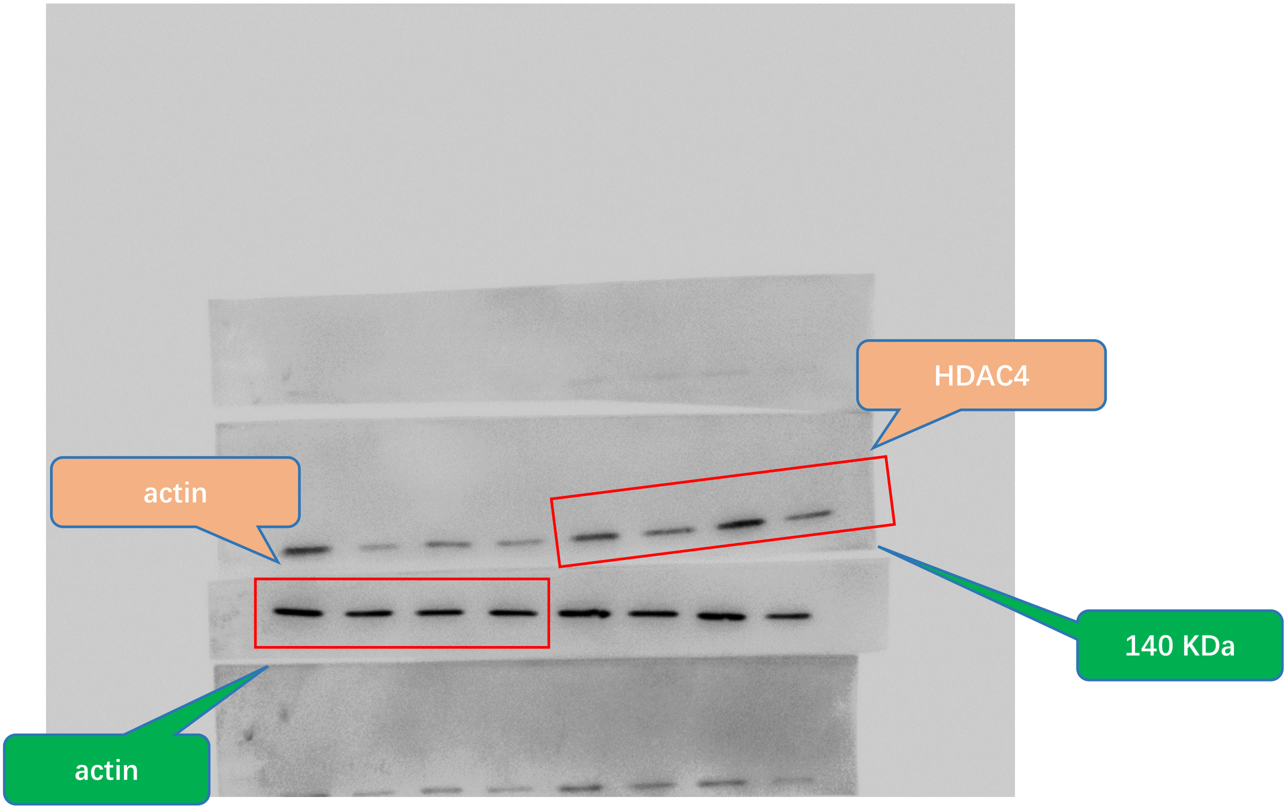

Supplement: Supplementary file 1 — Original data file_WB blots [file 41420_2022_1099_MOESM1_ESM.pdf]
