# Supplementary figures and images for: N6-methyladenosine (m6A) methyltransferase METTL3 regulates sepsis-induced myocardial injury through IGF2BP1/HDAC4 dependent manner
Source: Cell Death Discov. 2022 Jul 15;8:322. doi: 10.1038/s41420-022-01099-x (PMC9287338; doi:10.1038/s41420-022-01099-x)

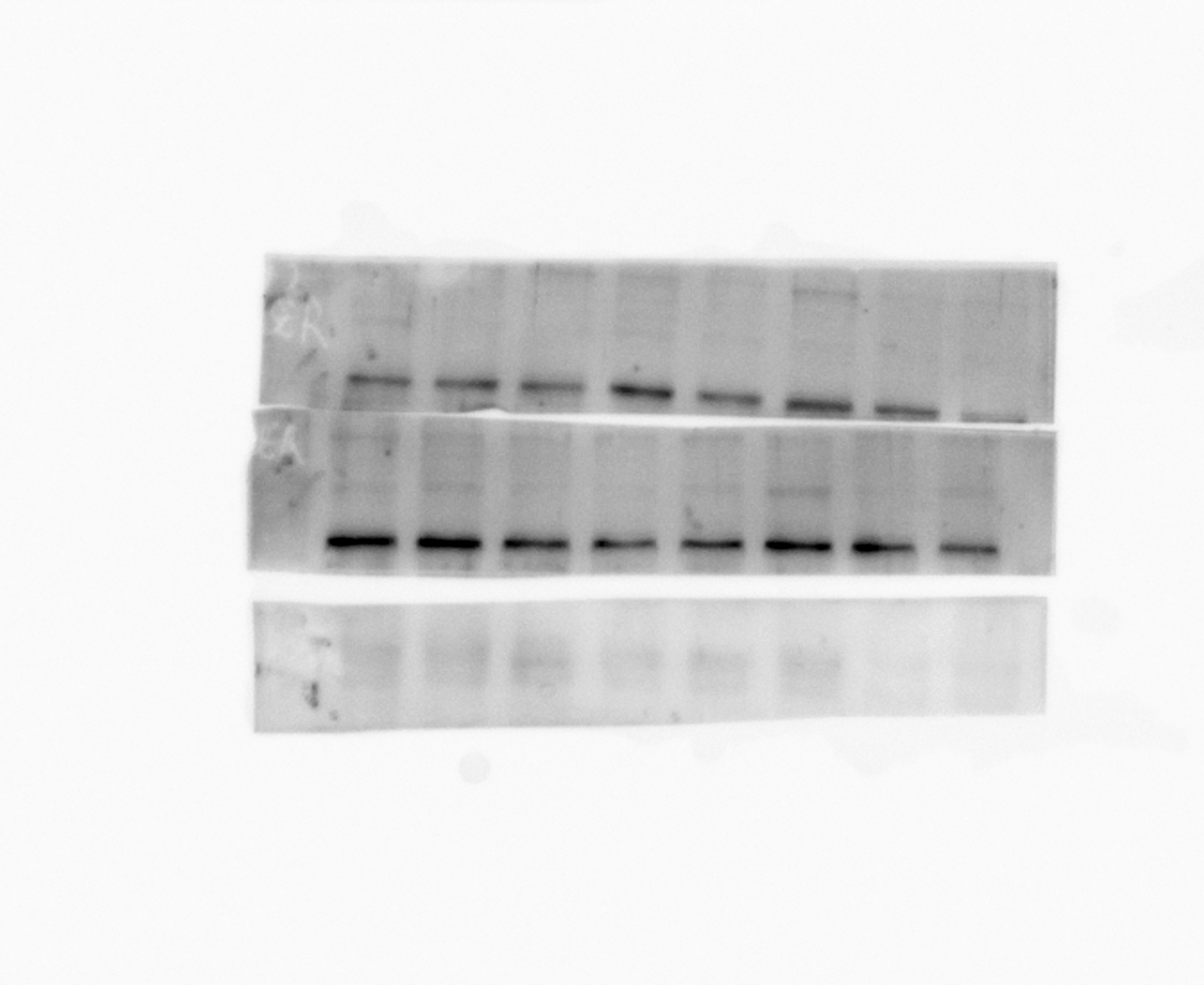

Supplement: Supplementary file 2 — Fig 1 Actin blot [file 41420_2022_1099_MOESM2_ESM.tif]

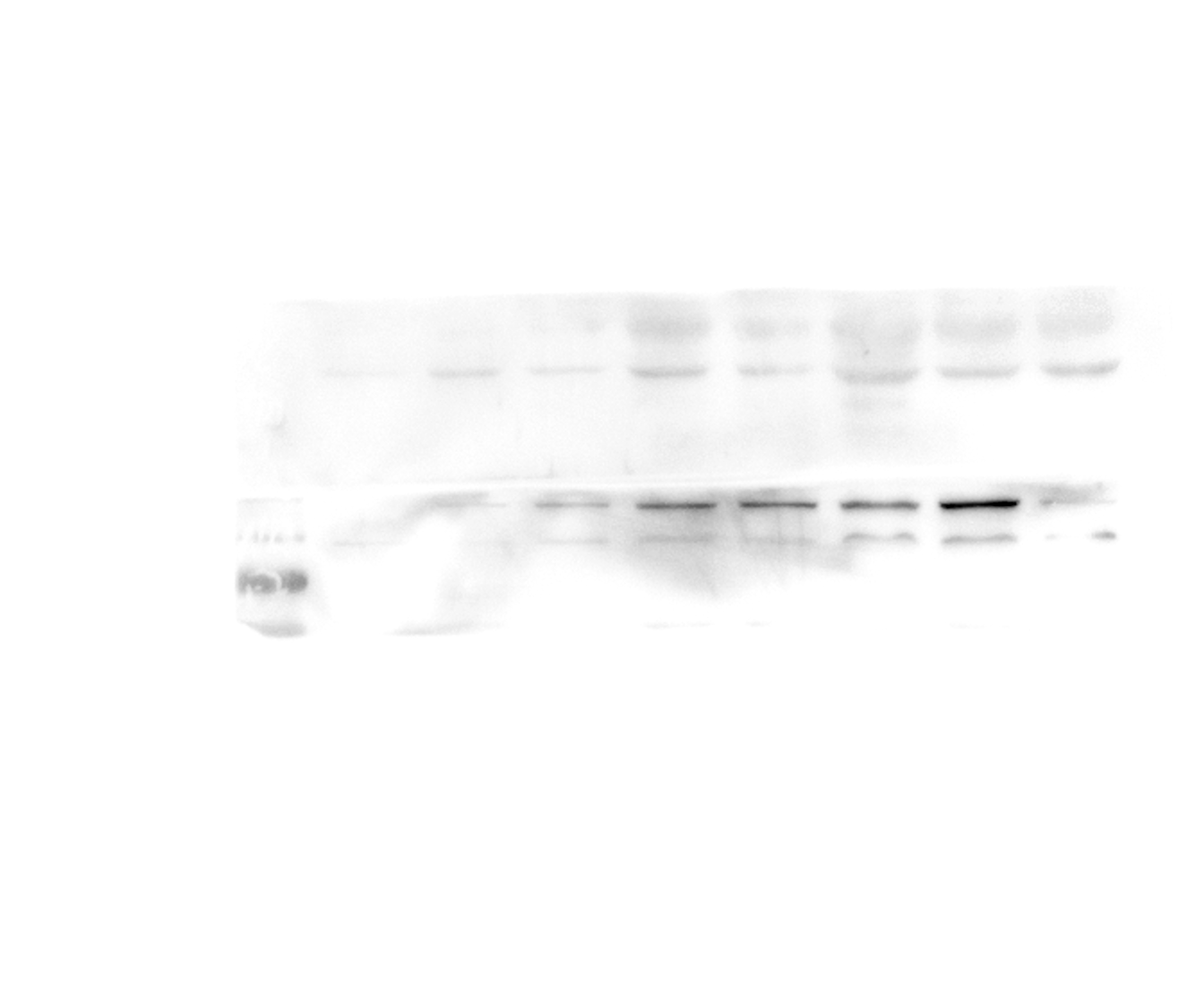

Supplement: Supplementary file 3 — Fig 1 METTL3 blot [file 41420_2022_1099_MOESM3_ESM.tif]

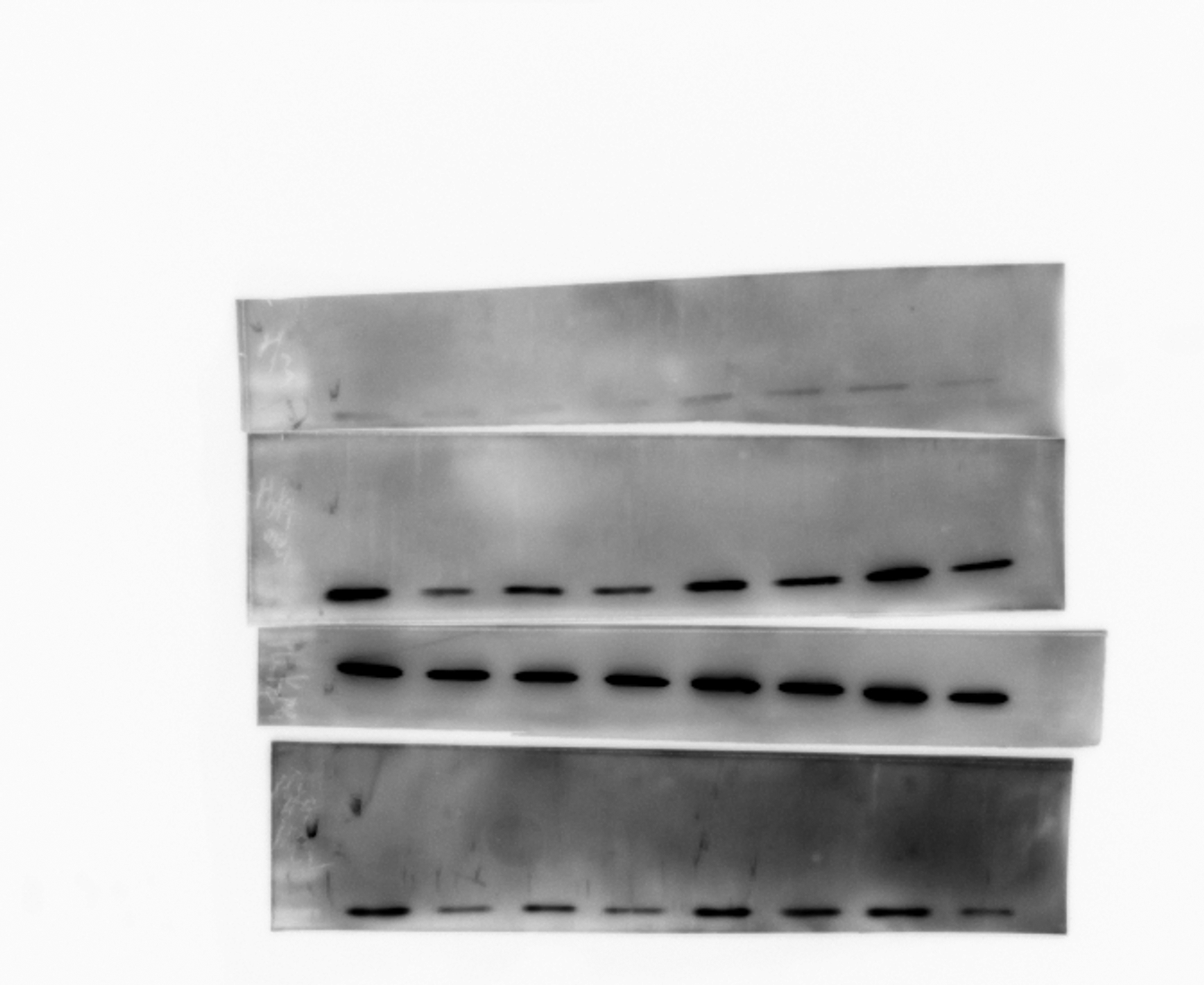

Supplement: Supplementary file 5 — Fig 2 METTL3 [file 41420_2022_1099_MOESM5_ESM.tif]

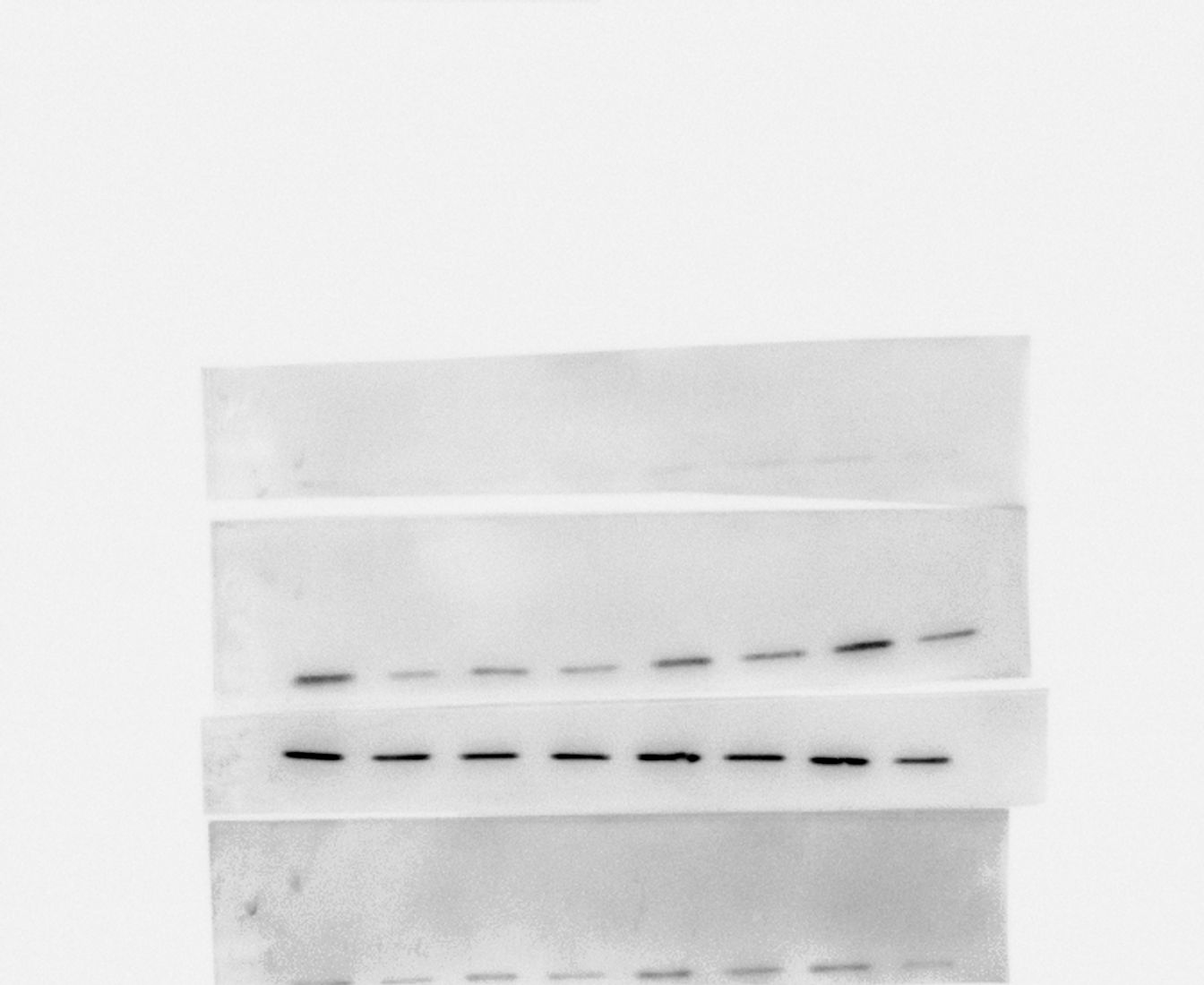

Supplement: Supplementary file 6 — Fig 5 [file 41420_2022_1099_MOESM6_ESM.tif]
