# Supplementary material for: N6-methyladenosine (m6A) methyltransferase METTL3 regulates sepsis-induced myocardial injury through IGF2BP1/HDAC4 dependent manner
Source: Cell Death Discov. 2022 Jul 15;8:322. doi: 10.1038/s41420-022-01099-x (PMC9287338; doi:10.1038/s41420-022-01099-x)
Supplement: Supplementary file 7 — Table S1 [file 41420_2022_1099_MOESM7_ESM.docx]

**Table S1**. Primers sequences for qRT-PCR and sequences of shRNA.

|  | Sequences |
| --- | --- |
| METTL3 | forward, 5’-TTGTCTCCAACCTTCCGTAGT-3’  reverse, 5’-CCAGATCAGAGAGGTGGTGTAG-3’ |
| IGF2BP1 | forward, 5’-GCGGCCAGTTCTTGGTCAA-3’  reverse, 5’-TTGGGCACCGAATGTTCAATC-3’ |
| sh-METTL3-1 | Sense, 5’-GCUGCACUUCAGACGAAUUTT-3’  Anti-sense, 5’-AAUUCGUCUGAAGUGCAGCTT-3’ |
| sh-METTL3-2 | Sense, 5’- GCAGGAACGTGGAGCTGGGCA-3’  Anti-sense, 5’-AAUCUUCUGAAGAUGCGGCTT-3’ |
| HDAC4 | forward, 5’- GGCCCACCGGAATCTGAAC-3’  reverse, 5’- GAACTCTGGTCAAGGGAACTG-3’ |
| GAPDH | forward, 5’- GGAGCGAGATCCCTCCAAAAT-3’  reverse, 5’- GGCTGTTGTCATACTTCTCATGG -3’ |
